# The future of robotic disassembly: a systematic review of techniques and applications in the age of AI

**DOI:** 10.3389/frobt.2025.1584657

**Published:** 2025-10-09

**Authors:** Soufiane Ameur, Mohamed Tabaa, Zineb Hidila, Mohamed Hamlich, Kaouter Karboub, Richard Bearee

**Affiliations:** 1 CCPS Laboratory, ENSAM-C, Hassan II University, Casablanca, Morocco; 2 Pluridisciplinary Laboratory of Research and Innovation (LPRI), EMSI Casablanca, Casablanca, Morocco; 3 LISPEN, Arts et Métiers Institute of Technology, Lille, France

**Keywords:** robotic disassembly, AI approaches, human robot collaboration, computer vision, systematic review

## Abstract

In today’s era of digital transformation, industries have made a decisive leap by adopting data-driven, robot-assisted disassembly solutions that cut cycle time and cost relative to labor-intensive manual tear-down. Thus, including robots not only improved production activities but also strengthened the safety measures that once the human operator was handling. Minimizing the impact of the human factor in the process means minimizing incidents related to it. The disassembly of Waste Electrical and Electronic Equipment (WEEE) poses complex technical, economic, and safety challenges that traditional manual methods struggle to meet. Thus, there is a need for a decision-making tool harmonized with human cooperation, in which Artificial Intelligence (AI) plays a pivotal role by providing financially viable solutions while ensuring a secure collaborative environment for both humans and robots. This review synthesizes recent advances in AI-enabled robotic disassembly by focusing on four main research areas: i optimization and strategic planning, ii human–robot collaboration (HRC), iii computer vision (CV) integration, and (iv) Safety for Collaborative Applications. A supplementary subsection is also included to briefly acknowledge emerging topics such as reinforcement learning that lie outside the main scope but represent promising future directions. By analyzing 62 peer-reviewed studies published between 2000 and 2024, the results identify how these themes converge, highlight open challenges, and map out future research directions.

## 1 Introduction

**FIGURE 1:**
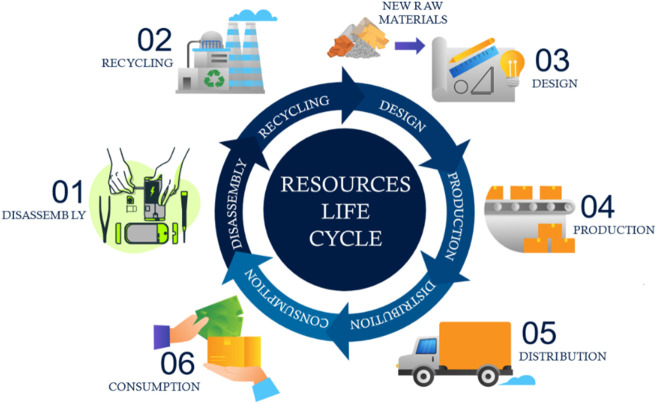
An abstract visualization of the life cycle of resources according to the circular economic business model. 01: Dismantling of the EOLP; 02: Recycling of materials; 03: New raw materials enter production during the design and manufacture of sub-components; 04: Production of the final product; 05: Distribution of the product to customers; 06: Consumption of the product.

Natural resources used in electronics cannot be regenerated, or at least not at the same rate at which they are consumed. The United States itself generated 500 million volumes of electronic waste between 1997 and 2007 (Kiddee et al., 2013). During that period, printed-circuit boards (PCBs) relied on costlier raw materials underscoring the imperative for resource stewardship and long-term sustainability (Perossa et al., 2023). Remanufacturing is defined as the process of bringing back a used product up to the level of its original equipment manufacturer (OEM), with the same warranty as an equivalent new product (Matsumoto and Ijomah, 2013). It has a major impact on preserving the environment, thanks to the use of recovered components which are then reassembled into remanufactured products, saving on raw materials as well as reducing production costs while at the same time reducing the impact on the environment. A visualization of the life cycle of resources according to the circular economic business is presented in Figure 1. The early adoption of such processes was driven by the need to repair, maintain or understand complex machinery. However, as products became more complex and the need to recover individual components expanded, the term ‘disassembly’ was introduced allowing valuable components to be extracted in a targeted manner, thereby facilitating efficient recycling and reducing the environmental footprint. The disassembly process represents the first phase in the remanufacturing cycle (Priyono et al., 2016). It is the reverse process in which a product is separated into its components and/or sub-assemblies by non-destructive or semi-destructive operations that damage only the connectors or fixings. If the process of separating the product is not reversible, this process is called disassembly (Vanegas et al., 2019). Within the resource life cycle, the disassembly process itself focuses on the extraction of sub-assemblies and individual components from end-of-life products (EOLPs) so that they can be reused/manufactured. Non-destructive disassembly refers to separating components without damaging them, enabling their reuse, remanufacturing, or recycling. While this preserves the integrity of individual parts, it does not necessarily allow for full reassembly of the original product and is therefore not always fully reversible. However, when it involves waste electrical and electronic equipment, the main obstacles to successful recycling (both technical and economic) include the difficulties associated with classifying and disassembling components. Manual operations are considered prohibitively expensive, and full automation is also rejected due to the lack of uniformity of discarded appliances and the exorbitant costs associated with traditional automation techniques (Alvarez-de-los-Mozos and Renteria, 2017a). Manual disassembly also causes safety problems which increase labor costs, representing the second most expensive item for a recycling plant (D’Adamo et al., 2016). Following the industrial revolutions, starting with the first industrial robot up to the advanced technologies of industry 4.0 and 5.0. Robots in industrial processes make industrial plants even more efficient, reducing errors and safety issues while improving both product stability and consistency. For this reason, one interesting solution consists of integrating robots into the disassembly process. The use of robots is increasing in remanufacturing systems which particularly improves the performance of disassembly lines. In remanufacturing systems, robots can be deployed in various roles ranging from fully autonomous execution of specific tasks to collaborative operations alongside human workers or other robots, with the flexibility to adapt to different task requirements. The main advantage of robotics is in the accurate and consistent performance of repeated tasks, such as on assembly lines. On the other hand, in the context of robotic disassembly which involved several uncertainties, a standard robot without any cognitive capacity for reasoning and logic will have serious limitations compared with the ability of a human being to disassemble an EOLP intuitively. To fully realize the potential of automated disassembly, it is essential to implement artificial intelligence approaches such as reinforcement learning (RL) alongside with computer vision systems capable of automatically identifying and locating such items or finding the most optimized path (Wegener et al., 2015).

This paper begins by presenting the background of robotic disassembly (Section 1), followed by the paper selection methodology (Section 2) used to identify relevant studies across four main areas, and then proceeds to a detailed analysis of selected studies (Section 3), which will be divided according to domain.

## 2 Background: robotic disassembly

Robots enable faster, more consistent extraction of reusable components from end-of-life (EOL) products. Robotic systems now replace manual disassembly techniques to make recovery of reusable materials from electrical and electronic products faster. Companies demonstrate this breakthrough in their recycling processes. In 2016, Apple introduced Liam and Daisy which demonstrated a treatment process of e-waste by disassembling an iPhone within minutes (Apple, 2024). Another example presented by CRG Automation deployed robotic systems to safely disassemble the M55 rocket, a chemical weapon containing nerve agents such as VX and sarin (James, 2023). Without forgetting many use cases such as e-waste or battery disassembly. Or their automated solution enabled precise handling and neutralization within high-risk demilitarization facilities (Allison, 2023; Fraunhofer IFF, 2025). Robotic disassembly gives manufacturers promising performance benefits that combine flexibility with profitability and safety protection along with positive environmental outcomes. Robots can also handle many different products as human operators. In addition, robots improve both labor savings, making remanufacturing more affordable. Using robotic disassembly helps with material reuse which lowers environmental effects (Zeng et al., 2022). Finally, robots can perform in unsafe areas and manage dangerous materials making human workers safe (Xu et al., 2021).

### 2.1 Collaborative approaches for disassembly

The existing e-waste management is confronted with a couple of issues: Manual disassembly process is expensive, and automated disassembly is complicated for virtually all types of legacy devices. The current approach is tackling the issues using a hybrid approach where robots and human operators collaborate (Alvarez-de-los-Mozos and Renteria, 2017b). This approach incorporates robotic and human operators to facilitate e-waste recycling with the use of the best system designs under ecosystems. The basics of this field include interactions between people and robots as well as other advanced constructs of human-robot collaboration, wherein the robot is endowed with the skills needed to work with people (Feil-Seifer et al., 2009; HRI, 2024). Human-centered collaborative robotics creates shared workspaces in which robots handle repetitive or hazardous operations such as manipulating irregularly shaped, toxin-laden components while humans provide real-time judgement. Analyzing e-waste disassembly therefore requires a concurrent examination of collaborative strategies and the enabling tool-chain.

**FIGURE 2:**
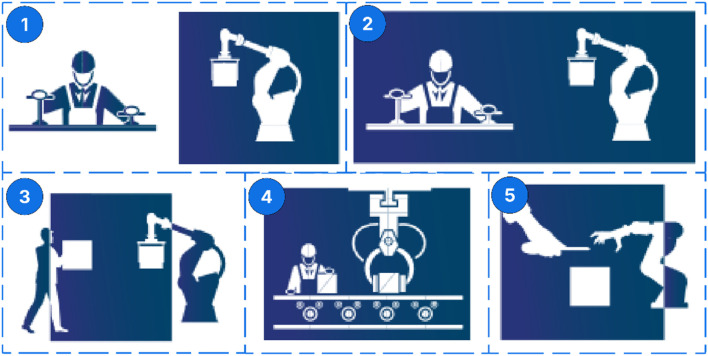
Collaborative approaches in robotic disassembly (1. Robot Cell, 2. Coexistence, 3. Synchronized, 4. Cooperation, 5. Collaboration).

A specialized robotic cell for disassembly functions as a system that uses PLC-controlled robots to execute disassembly tasks under remote monitoring conditions (Dawande et al., 2005). Product separation and hazardous element removal procedures through recycling operations occur frequently with this technology to achieve layout disassembly goals. Robotic cells integrate built-in security and environmental awareness with custom disassembly techniques but require human agency to complete tasks which need direct assistance. Human Robot Coexistence operations at disassembly sites yield challenging situations together with fresh prospects for site management. People working alongside robots achieve versatile disassembly task integration by having robots complete excessive or dangerous procedures while humans handle decision-focused activities (Magrini et al., 2020). Today’s industries depend heavily on robotic technology for safety purposes because these machines safely handle toxic materials alongside sharp objects. Technical detection systems working with flexible robotic elements alongside cautious safety protocols help reduce exposure risks to create optimized work environments for concurrent human-robot disassembling operations (Magrini et al., 2020). Humans and robots perform their work simultaneously in the same space through a coordinated disassembly method (Zhuang et al., 2019). During synchronization the human workers share the space with robotic systems through parallel task execution that maintains individual work domain separation. Human operators first remove screws from the workpiece, creating space for robotic extraction operations that achieve highly precise results. This integrated approach combines robotic processing elements alongside human capabilities to increase complex disassembly performances through enhanced accuracy rates and operational speed increases. When humans work with robots in identical workspace areas independent roles integrate as part of collaborative tasks. When companies adopt collaborative systems, they achieve effective workspace division between parallel assignments without sacrificing production objectives (Váncza et al., 2011). After robot systems break down single components human operators check these pieces to verify component condition before permitting additional disassembly operations. The current advanced technology enables several operations to execute simultaneously by forcing robotic employees to stay regardless of preceding work completion so tasks function without interruption. The methodology enables flexible operations within complex disassembly systems by implementing its collaborative process. The core technique behind robot-human operator collaboration allows teams to work together in both safe and optimized conditions (Ameur et al., 2024). Figure 2 illustrates the collaborative approaches in robotic disassembly.

### 2.2 Challenges and difficulties

However, robotic disassembly of WEEE introduces a wide range of associated challenges that make automation particularly difficult when compared to assembly processes. Among the most critical obstacles is the need to operate in dynamic environments in which variability in product orientation, product condition and component integrity can interfere with fixed robotic routines. Contrary to structured industrial tasks the disassembly often takes place in unpredictable spatial and material conditions which require real-time detection and adaptation. And there are no uniform end-of-life conditions as products reaching the end of their life may be damaged at some stage, incomplete or severely worn (Hohm et al., 2000). Different versions of products, user modifications may result in different internal configurations which limit the effectiveness of predefined CAD-based trajectories or fixed motion sequences (Bogue, 2019). As a result of this unpredictability, the cognitive and mechanical requirements of robotic systems increase. It also involves a variety of tooling requirements (Kernbaum et al., 2009). As opposed to repetitive assembly, the disassembly process often requires several operations on a single product. This may involve unscrewing, cutting, breaking, heating or lifting components. Each of these operations may require a different tool head and actuation force and level of precision. This requires reconfigurable end-effectors and tool change mechanisms that offer multiple functions while maintaining cycle time and safety (Poschmann et al., 2021; Karlsson and Järrhed, 2000). The coordination of these systems is even more complex when it comes to seamless automation. Perception, as well as decision-making and motion planning, must be tightly synchronized in an integrated way, especially in cluttered or constrained environments. Robots must be able to make decisions regarding the disassembly sequence and execute high-precision movements, all in real time. In collaborative scenarios, the coordination between human and robotic agents becomes even more critical so robust interaction protocols and safety mechanisms are required. Other systemic barriers can include designs that are not intended for disassembly, where products are manufactured to be compact and tamper-proof with strong adhesives and welded joints or concealed fixings. Such features make automated disassembly technically unfeasible or economically inefficient (Huang et al., 2021). The environmental and regulatory constraints associated with WEEE add to the complexity since robots need to securely extract toxic components such as lithium batteries or mercury lamps while maximizing the recovery of valuable materials such as rare earth elements. In addition, the availability of datasets and standardization remain major obstacles. While assembly is documented and often standardized at every stage, the disassembly process has no guidelines or detailed labelling. This limits the ability to form intelligent systems or to generalize robot behavior across product types.

## 3 Paper selection methodology

**FIGURE 3:**
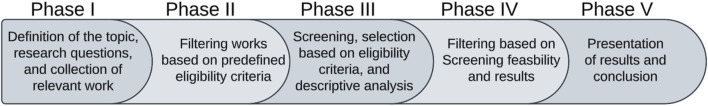
The five Phases of the Paper Selection Methodology as adapted from Moher et al. (2009).

A systematic review selection framework was developed specifically to conduct thorough research on robotic disassembly processes and the integration of AI methods. This study follows preferred reporting items for systematic reviews and meta-analyses (PRISMA) guidelines as defined by Moher et al. (2009) through a five-stage framework (Figure 3). Phase I begin by developing an explanation of the topic then selecting research questions before retrieving publications through multiple information platforms. The second phase of methodology implements predefined eligibility criteria to refine the initial study pool which helps researchers locate appropriate and researched-based documents. During Phase III an extensive screening protocol integrates eligibility verification with descriptive investigations of approved reviews. With Phase IV researchers examine the chosen studies to discover feasibility levels and confirm study objectives match. Results from this research investigation present critical findings in Phase V. The structured systematic process safeguards the scientific validity of the review through detailed outcomes which researchers can easily understand.

### 3.1 Search strategy

The research query covered the four major robotic disassembly domains through precise search terms which included optimization approaches alongside strategic planning methods denoting high-level, offline decisions that structure the entire disassembly system and human-robot collaboration systems. The research included search strings that combined “Robotic disassembly” with “Disassembly automation” to find general robotic disassembly studies and “Human-robot collaboration” with “Collaborative robots” to identify specific Human-Robot Collaboration research. The analysis included advanced technology searches with combinations of “Computer vision disassembly” OR “AI vision systems in robotics” to examine robotic applications that merged vision systems with artificial intelligence. Research examining safety practices in robotic disassembly is covered through search terms that include “Safety in robotic disassembly” OR “Human safety in collaborative robotics” (Table 1). A methodical searching system enables the review to identify an extensive collection of research documents which accurately represents the current body of knowledge.

**TABLE 1:** Search string and operators.

Topic	Search string and operators
Robotic Disassembly	“Robot disassembly” OR “Cobot disassembly” OR “Disassembly automation” OR “Industrial robotic disassembly” OR “Automated reverse engineering”
Optimization and Strategic Planning	“Optimized Robotic disassembly” OR “Robotic Optimization disassembly” OR “Strategic Planning disassembly” OR “Efficient Robotic Disassembly” OR “Robotic disassembly line balancing problem (RDLBP)” OR “Disassembly sequencing” OR “Path optimization in disassembly”
HRC	“Human-robot collaboration” OR “Collaborative robots” OR “HRC disassembly” OR “Human-robot interaction in disassembly” OR “Robot-assisted human disassembly” OR “Shared control in robotics” OR “Hybrid disassembly systems”
CV Integration	“Computer vision disassembly” OR “Machine vision in robotics” OR “Vision-guided robotics disassembly” OR “Image processing for disassembly tasks” OR “Object detection in robotics” OR “Vision-based disassembly systems” OR “AI vision systems in robotics”
Safety	“Safety in robotic disassembly” OR “Disassembly safety protocols” OR “Human safety in collaborative robotics” OR “Safety optimization in robotics” OR “Robot safety systems” OR “Hazard prevention in disassembly processes” OR “Standards for safe human-robot collaboration”

The research period encompassed publications from 2000 to 2024 (Table 2) to capture advancements in robotics and artificial intelligence and industrial disassembly methods during the last 24 years. The analysis included only publications written in English to ensure uniformity throughout data extraction and interpretation. The research included only peer-reviewed articles and conference proceedings alongside industrial standards (such as ISO) and technical reports that provided full text access through online distributors or direct access. The research excluded materials which did not meet peer-review standards or used non-English text or focused on unrelated robotic disassembly or AI applications. The analysis excluded research papers that analyzed assembly operations alone without discussing reverse engineering or disassembly work. The established criteria allowed researchers to select reviews which directly focused on review objectives while preserving scientific standards.

**TABLE 2:** Reviewing protocol.

Item	Description
Time Period	Publications from 2000 to 2024
Language	Only English publications were considered to ensure consistency in data extraction
Availability	articles available online as full text directly or via distributor
Publication type	Journals, conference proceedings, industrial standards (e.g., ISO), technical reports
Exclusion criteria	Studies not peer-reviewed, non-English papers, articles unrelated to robotic disassembly, AI applications or the four topics, and studies focusing on assembly

### 3.2 Paper selection

**FIGURE 4:**
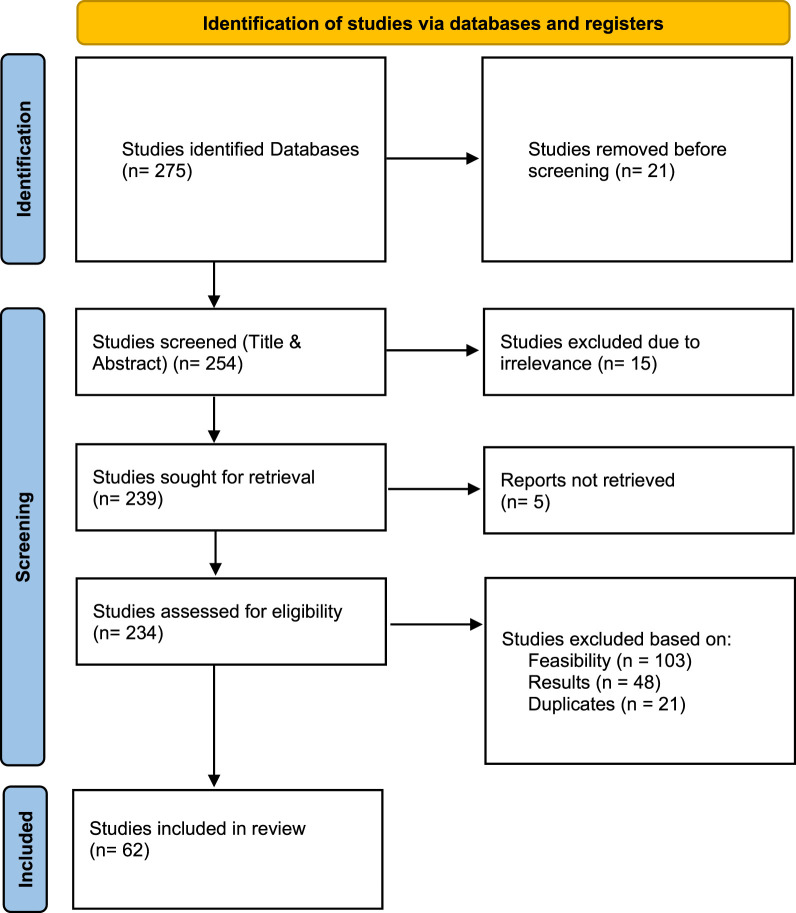
PRISMA flow diagram of studies selection procedure.

To strengthen the examination process, the PRISMA flow diagram was used (Figure 4). In this way, a systematic and transparent evaluation of the literature is guaranteed, reinforcing the reliability of the findings and comparison. The inclusion and exclusion criteria were applied in distinct stages as follows:

#### 3.2.1 Title and abstract screening

The database search revealed 275 publications, which included 198 from Google Scholar, 47 from Scopus and 30 from Web of Science. Our analysis has eliminated 21 duplicates and evaluated 248 separate research articles (Table 3). Those publications were selected by the research team through a title and abstract evaluation process in order to find their relevance to the research topic. Evaluation included elimination of research that did not focus on robotic disassembly techniques or artificial intelligence applications.

**TABLE 3:** Initial set of studies found in relevant databases.

Databases	Final set of studies
Google Scholar	198
Scopus	47
Web of Science	30

#### 3.2.2 Full-text screening

A thorough review of full texts applied to 254 remaining articles. The research excluded 192 articles throughout this stage for either being unfeasible to implement or showing insufficient data or lacking alignment with the research context which included studies unrelated to disassembly systems or human-robot collaboration or safety. A thorough examination of 62 articles during this phase resulted in a final selection of articles for comprehensive research.

#### 3.2.3 Sorting based on content type

The final 62 articles received content-based categorization that focused on the review’s four main themes including optimization strategies and human-robot collaboration and computer vision integration alongside safety for collaborative applications. These articles served as the base for descriptive research and content analysis that followed in the review process.

### 3.3 Content analysis and classification

**FIGURE 5:**
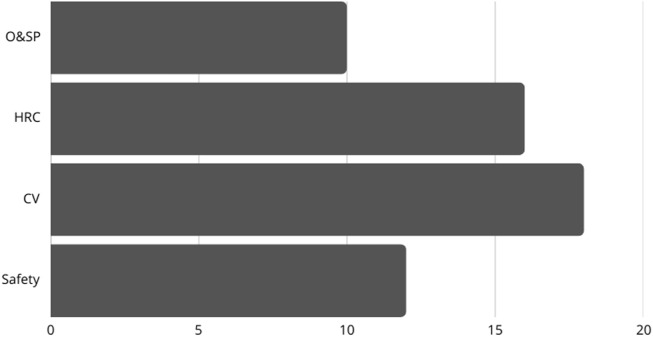
Topic Distribution of review.

This classification system defines the research spaces within robotic disassembly studies (Figure 5). Through optimization and strategic planning (O&SP) techniques developers create essential algorithms that optimize both sequencing planning processes and resource allocation effectiveness. Ergonomic system designs which enable HRC produce spaces that are safer and more productive for shared operations. Advanced visual systems integrated through computer vision technology enable robots to work with greater precision when detecting objects and sequencing disassembly operations. Safety represents an ongoing necessity because risk-minimizing systems need appropriate protocols to operate with autonomous systems and collaborative systems alike. Research within robotic disassembly studies shows how artificial intelligence systems can solve various problems by linking different domains.

**FIGURE 6:**
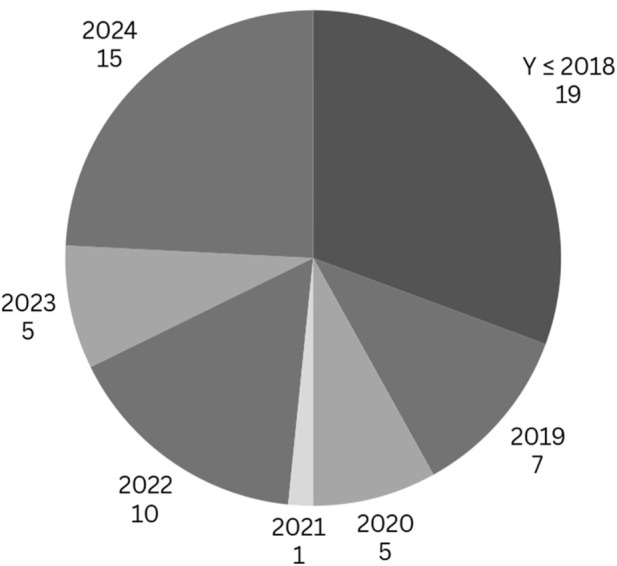
Pie chart of Year Distribution.

Figure 6 demonstrates strategic selection toward current discoveries while including essential studies in robotic disassembly and Artificial Intelligence research. Post-2019 scholarly work dominates the selection since it demonstrates advanced robotic disassembly techniques and Artificial Intelligence applications. Recent studies reveal new understanding about the operation of reinforcement learning systems together with collaborative robots and computer vision software. A selection of 19 groundbreaking papers originating from before 2018 serves as foundational material for subsequent investigation.

This research organizes its sources into three main categories including journal articles and conference papers and others for clear understanding of resource analysis. Journal articles present extensive examinations of field knowledge that deliver basic and advanced understanding. Conference papers highlight contemporary innovations and developmental progress which take place at prominent industrial events. The “Other Sources” category in Table 4 contains industry reports and white papers and industrial standards which strengthen the practical value of the study. The table provides an overview of source categories together with their assigned reference counts.

**TABLE 4:** Categories of the selected papers.

Categories	Number
Journal Articles	43
Conference Papers	11
Other Sources	8

## 4 Detailed analysis of selected studies

Artificial intelligence has emerged as an essential tool for tackling the complexity inherent in robotic disassembly systems (Poschmann et al., 2020). Within WEEE recycling, combining different types of robots such as industrial high-precision arms with flexible collaborative robots can broaden task coverage but also increases integration challenges due to different control interfaces, communication protocols and tooling requirements. AI overcomes these challenges by providing adaptive perception, decision-making and control capabilities that enable robots to navigate complex product geometries and identify components and also perform disassembly steps with greater accuracy and efficiency. Conceptually, AI refers to the integration of human-like reasoning and learning into machines. Machine learning forms the core to enable systems to derive patterns from data and automatically build analytical models, while deep learning takes advantage of multi-layer neural networks to model complex relationships in visual, spatial or sequential data (Saadat et al., 2022). Generative AI extends these capabilities to content creation, which, in the disassembly domain, can support tasks such as synthetic data generation for training vision systems (Sætra, 2023). The following sub-sections provides a detailed analysis of selected studies grouped into four key research domains that structure the current landscape of AI-enabled robotic disassembly: optimization and strategic planning, human–robot collaboration, computer vision integration, and safety standards. These areas were identified as the most recurrent and impactful across the reviewed literature. A final subsection highlights supplementary trends, including emerging approaches such as reinforcement learning, which while outside the main scope, indicate promising future directions.

### 4.1 Optimization and strategic planning

The strategic planning along with navigation optimization of robotic systems relies on AI algorithm execution (Chen et al., 2021). This capability allows the robot to effectively determine the most straightforward path toward dismantling without harming significant components and while reducing operational mistakes. Guo et al. (2023) implemented a dual-agent approach using Deep Q-learning (DQL) and RL to create decision frameworks which enhanced state exploration efficiency and calculation speed. Lee et al. (2022) established a computational framework for HRC that combines safe human conditions with resource limitations to optimize complete disassembly durations within defined security boundaries. Qu et al. (2023) employed RL together with neural networks and actor-critic modeling to teach robots how to extract bolts from door chain grooves resulting in less than 1 mm of clearance between components. The objective of this work was to improve robotic disassembly capabilities through skill transfer and training capabilities. Çil et al. (2020) utilized Ant Colony Optimisation (ACO) along with Genetic Algorithm (GA) and Random Search (RS) algorithms for comparative analysis. ISIACO represents a proposed solution which uses multiple algorithms to optimize disassembly line balancing while incorporating the best elements of existing solution approaches to achieve improved performance. The study by Laili et al. (2022) shows how backup actions boost disassembly sequence planning reliability during automation system failures. The research presents three backup action types and introduces a new approach to disassembly planning along with the proposed DS-MOEA solution method. A two-pointer detection system combines with interference matrix technology to determine extractable components from subassemblies. The algorithm surpasses conventional methods by constructing optimal sequence plans while attaining superior completion results through performance enhancement. The research by Hartono et al. (2022). developed a robot planning model which determines disassembly product sequences to maximize profits alongside saving energy and reducing greenhouse gas emissions. A computational model implementing the bee algorithm takes inspiration from bees’ natural food search behavior. The algorithm functions to enhance the efficiency of disassembly plans. The Bees algorithm functions to determine both optimal recovery options and associated disassembly information. Ramírez et al. (2020), Covers how to use optimisation technologies and methodologies, including hybrid cellular automata (HCA) and GA, to solve the disassembly sequencing problem. The paper also provides a detailed description of the customisation of various operations to solve the disassembly problem, including the size of the population, initialisation, crossover, mutation operators also as stopping criteria. The paper shows the results of the ideal solution for different algorithms, HCA, GA and CGA, with respect to fitness value and execution time. Lambert (2003), outlines the latest research into the modelling, scheduling, and applications of the disassembly process. Zhou et al. (2019), gave a paper where disassembly sequence planning (DSP) methods are introduced from the point of view of disassembly modelling and disassembly planning methods. The paper describes the characteristics associated with different DSP methods as well as identifying future directions for DSP. Zhang et al. (2014) showed a parallel disassembly fuzzy-rough set mapping model that has been implemented to obtain the ideal parallel disassembly sequence. Some recent advances proposed by Ming et al. (2019) address the balancing of multirobotic disassembly lines with uncertain processing times using multirobotic systems and stochastic task processing. On the same theme, Xu et al. (2023) have explored a discrete brainstorming multi-objective optimizer, this was done for balancing robotic disassembly lines in the event of disassembly failure and product variability. The comparative results of these works are synthesized in Table 5.

**TABLE 5:** Comparative table of algorithms used in optimization and strategic planning.

Authors	Technologies/Algorithm	Industry/Application
Guo et al. (2023)	Q-Learning - Deep Q-Learning	Disassembly Lines
Lee et al. (2022)	Disassembly Sequence Planning (DSP)	Task Planification
Qu et al. (2023)	Deep Deterministic Policy Gradient (DDPG) - Delayed Updates - Adam Optimizer	Robotic disassembly operations
Çil et al. (2020)	Mixed-Integer Linear Mathematical Model, Ant Colony Optimization	Robotic disassembly line balancing problem (RDLBP)
Laili et al. (2022)	Dual-Selection Multiobjective Evolutionary Algorithm (DS-MOEA)	Sequence Planning
Hartono et al. (2022)	Bees Algorithm	Disassembly plans
Ramírez et al. (2020)	HCA, GA, CGA	Disassembly plans
Lambert (2003)	Component-oriented, Product-oriented, Hierarchical tree, Reverse logistics approaches	Sequence Planning
Zhou et al. (2019)	Nature-inspired heuristic algorithms (NIHA) - Linear Programming Methods (LPM) - Rule-Based Methods (RBM) - Stochastic Simulation (SSI)	Sequence Planning
Zhang et al. (2014)	Fuzzy-rough set mapping	Parallel disassembly – Sequence Planning
Ming et al. (2019)	Mixed-Integer Programming Model, Task Precedence Diagram	Multi-Robotic Disassembly Line
Xu et al. (2023)	Multi-Objective Discrete Brainstorming Optimizer	Robotic Disassembly Line Balancing

### 4.2 Human-robot collaboration

Thanks to human-robot collaboration, disassembly tasks have become more efficient and flexible. Such collaborative action optimises the use of resources by giving repetitive or physically demanding tasks to robots, while leveraging human problem-solving and adaptability skills. Thanks to advanced sensors and safety features, robots can work next to human operators. HRC’s adaptability offers major advantages for disassembly, as it enables rapid adaptation to different types of products as well as materials. In addition, working in this collaborative mode makes it easier to improve human skills, as robots help to stabilise components or carry out complex tasks. Hjorth and Chrysostomou (2022), Matheson et al. (2019) explores in a literature review, different technologies and standards related to human-robot collaboration in disassembly processes. Also, they focus on the technology and approaches used in human-robot collaborative disassembly systems. Kay et al. (2022), concluded that the optimum disassembly solution for an EV battery pack/module should be a human-robot collaboration, where the robot can efficiently make cuts on the battery pack, allowing the technician to quickly sort out the battery parts and remove any plugs or connectors that the robot is having trouble with. Li et al. (2018), have carried out research to overcome the challenges presented by the flexibility and reconfigurability of processing variable-sized components from electric vehicles, offering a robotized disassembly approach to boost value recovery and reduce environmental impact. Chen et al. (2014), describe a comprehensive state diagram for training a robot for a new bit position. With this approach, the robot can return several times to its initial position using joint control, thus improving the accuracy of the bit approach. The paper also offers some valuable insights into the development of robotic systems for unscrewing in disassembly processes, responding to the need for adaptability and flexibility within industrial automation. Chu and Chen (2023), present the results of a mathematical model which calculates the completion time under different conditions, and compare the performance of various optimization algorithms. Results reveal that the suggested approach achieves a reduced completion time while guaranteeing the sustainability of all disassembly sequences. One case study by Huang et al. (2020) shows a two-finger gripper KUKA LBR iiwa robot being employed to separate press-fit components, making use of active compliance monitoring along with impedance monitoring for safe and flexible interaction with human operators. Prioli and Rickli (2020), developed a cyber-physical architecture which uses human-robot interaction with collaborative robots (Cobots) to form a flexible automated disassembly system. The project aimed to solve the problem of executing large-scale disassembly operations which addresses uncertain end-of-life product conditions to support recycling and remanufacturing. Li et al. (2020) developed a control method which combines torque and position monitoring features with active compliance to unlock hex screws by using collaborative robots to improve end effector and screw head engagement success rates. Ding et al. (2019) proposes a knowledge graph-based system which enables human-robot collaboration during disassembly operations. In order to lower the downtime and disassembly cost, Wu et al. (2022) have proposed a study on a multi-objective optimization model to be implemented in human-robot collaborative disassembly extracting electric vehicle battery modules. In addition, a disassembly cell was designed by Huang et al. (2021) using active compliance and tactile sensing, making accurate human-robot interaction of complex elements such as automotive turbochargers. In Table 6, an overview of the referenced approaches.

**TABLE 6:** Comparative table of technologies/algorithms used in HRC.

Authors	Technologies/Algorithm	Industry/Application
Hjorth and Chrysostomou (2022)	Literature Review on HRCD (Human-Robot Collaborative Disassembly)	Industrial environments
Matheson et al. (2019)		Manufacturing applications
Kay et al. (2022)	Linear Quadratic Regulator (LQR) - Batch Least Squares Estimator - State Space Representation	Battery Modules
Li et al. (2018)	Case Study Product Analysis - Material Recovery Evaluation	Electrical vehicles
Chen et al. (2014)	Teaching by Demonstration - Collaboration Strategies	Unscrewing
Chu and Chen (2023)	Hybrid particle swarm optimization with Q-learning algorithm (HPSO_QL) - Particle swarm optimization (PSO) - Q-learning	Power batteries
Huang et al. (2020)	Cartesian impedance controller	Press-fitted components
Prioli and Rickli (2020)	Cobots	Critical Materials
Li et al. (2020)	Spiral search strategy	Unscrewing
Ding et al. (2019)	Knowledge graph - Knowledge base	Product disassembly
Wu et al. (2022)	Multi-objective optimization for disassembling waste power battery modules in a human-robot hybrid mode	Battery disassembly
Huang et al. (2021)	Active compliance, operator touch, and position control for a disassembly cell with complex geometries	Automotive Turbocharger

### 4.3 Computer vision integration

The combination of robotic devices with computer vision technology has transformed multiple industrial processes with robotic disassembly standing out as an exceptional application. Robotics-based disassembly operations require product or equipment extraction through computer vision methods which deliver unique advantages to this challenging process. By giving robots observation and environmental perception capabilities the accuracy levels together with operational speed and safety conditions of disassembly operations increase significantly. The accuracy and speed of robotic component identification improves when robots employ multiple product-specific detection models to update their environmental feature understanding. Research shows convolutional neural networks (CNN) (Chua, 1997) produce high image classification accuracy because they learn complex image data patterns, but their implementation demands big data sets and extensive computational resources. The YOLO (You Only Look Once) network stands apart through its real-time processing features and object detection speed of one pass through detection which benefits time-sensitive applications but demonstrates below-average efficiency in small-object scenes and cluttered scenes (Jiang et al., 2022). Region-based CNNs (R-CNNs) demonstrate top accuracy for detection and segmentation in challenging environments yet their high processing needs require extended computation times (Liu et al., 2016). The Single Shot MultiBox Detector operates at a higher speed than R-CNN while maintaining moderate accuracy levels between the two approaches. The Real-time applicability domain matches this approach. Fast R-CNN took up an objective like that mentioned above: The researchers aimed to make the initial R-CNN faster without compromising its precision (Zhang et al., 2018). SSD operates more rapidly than YOLO but slower than both detection models. Faster R-CNN delivers improved speed and accuracy in detecting small objects although its computational requirements surpass those of YOLO and SSD. By offering high precision capabilities RetinaNet detects objects of all sizes through a learning process that remains straightforward and efficient in terms of computational resources (Girshick, 2015). The Mask R-CNN enhances Faster R-CNN through the addition of instance segmentation to object detection while maintaining exceptional precision yet requiring a significant increase in processing time and computational power. These models work together to boost robotic disassembly operations which results in advanced automation efficiency (Vuola et al., 2019). To complement this discussion, Table 7 outlines the synthesized characteristics of prior works.

**TABLE 7:** Comparative table models used in computer vision.

Model	Key applications	Strengths	Weaknesses
CNN (Chua, 1997)	Component identification, defect detection	- High accuracy in image classification and object detection - Ability to learn complex patterns	- Requires large datasets - High computational cost
You Only Look Once (YOLO) (Jiang et al., 2022)	Real-time object detection	- Real-time processing - Single pass for object detection	- Lower accuracy for small objects - Less effective for highly cluttered scenes
Single Shot MultiBox Detector (SSD) (Liu et al., 2016)		- Faster than R-CNN - Balances speed and accuracy	- Lower accuracy compared to R-CNN for small objects
R-CNN (Zhang et al., 2018)	Object detection and segmentation	- High accuracy in object detection and segmentation - Effective for complex scenes	- Slower processing speed - High computational requirements
Fast R-CNN (Girshick, 2015)		- Faster than R-CNN- High accuracy in object detection and classification	- Still slower than YOLO and SSD - High computational cost
RetinaNet (Wang et al., 2019)		- High accuracy - Efficient in detecting objects of varying sizes	- Higher computational cost - Complex training process
Mask R-CNN (Vuola et al., 2019)		- Adds instance segmentation to object detection - High accuracy in detecting and segmenting objects	- Slower processing speed - High computational requirements

Brogan et al. (2021), presents deep learning methods and computer vision technology for automatic screw detection specifically designed for maintenance and disassembly operations. Researchers present different models along with their detection results for screws and objects utilizing both Average Accuracy (AA) and Frames Per Second (FPS) metrics. A training pool of 900 original images with 12.3 MPx resolution supported development and testing occurred across three distinct image sets containing 90 images each. The Tiny-YOLO v2 DL object detection system functioned as the testing model of choice. The paper by Vongbunyong et al. (2013) provided an extensive study of process monitoring for disassembly tasks through vision-based cognitive robotics systems along with oversight structures and decision pathways for achieving target objectives. Through IndiGolog’s programming platform a rule-based reasoning system operates an algorithm to minimize the evaluation space needed to execute operations at specified points during disassembly. The system maintains an execution loop until the desired goal state becomes reality. The system developed by Alvarez-de-los-Mozos E and team implements body tracking together with facial recognition with color segmentation to compute hand positioning for humans (Alvarez-de-los-Mozos and Renteria, 2017a). The development of human-robot interaction depends significantly on these processes. Through hand gestures combined with vocal instructions people can teach robots to understand specific operational settings. Mangold et al. (2022). have developed an adaptable system that performs quick screw head detection and classification in automated disassembly processes for reconditioning with robotic adaptability to diverse workpiece types. Using a 1280 × 1024 pixel monochromatic camera equipped with a 6 mm lens mounted on the robot arm to provide a hand-eye camera system, object detection architecture YOLOv5 is employed to locate and classify the screws within the perceived images. The dataset consists of 550 images, among which six categories of screw heads at different sizes. Deng et al. (2024), proposed an approach that aims to provide efficient and highly accurate real-time exposure control of vision-based robotic disassembly processes in difficult lighting conditions. It consists of three major modules: the region-of-interest (ROI) extraction module, along with the ROI quality assessment module in addition to the exposure time prediction module. Based on a deep learning-based object detection model, YOLOv5, the ROI extraction module can extract ROIs out of images captured using a variety of hypothetical lighting conditions. Deep learning with YOLOv5x performed better than conventional image processing techniques when identifying e-waste laptop parts as described by Bassiou et al. (2021). The experiment utilized Hasty. AI to label images of laptops with open or closed lids from a curated dataset. Yildiz et al. (2020) devised a visual perception system through deep learning and point cloud processing for automated hard disk (HDD) computer disassembly to achieve accurate gap detection. The researcher proposed a system for screw detection and localization in waste electronic products. The circular shapes that define screws as fundamental elements will be identified through Hough transform applications leading to a classifier which uses positive and negative training data examples. Using a dataset of over 10,000 samples, the performance of the screw classifiers is measured, and the two best-performing classifiers are combined into an integrated model (Yildiz and Wörgötter, 2019), Rehnholm (2021), concentrates specifically on two object detection approaches, pattern matching and CNNs, to benchmark how they perform in the task of dismantling electric vehicle batteries in order to find the optimal solution in terms of accuracy, recall performance as well as time consumption. A contrastive transfer learning framework (PLURAL) has been proposed by Biehler et al. (2023), which improves defect detection in 3D point clouds by using domain-invariant features. Zheng et al. (2022) have applied PointNet with the aim of identifying mechanical parts from 3D scans and facilitating robotic disassembly of complex automotive systems. The detailed characteristics of the cited contributions are organized in Table 8.

**TABLE 8:** Comparative table of technologies/algorithms used in CV integration.

Authors	Technologies/Algorithm	Industry/Application
Brogan et al. (2021)	Tiny-YOLO v2 - Deep Learning Models	Robotic disassembly
Vongbunyong et al. (2013)	Haar classifier - Golog Programming Language	Disassembly operations
Alvarez-de-los-Mozos and Renteria (2017a)	RGB-D Kinect Sensor for Human	E-waste management
Mangold et al. (2022)	Eye-in-hand vision system - YOLOv5	Screw Head Detection
Deng et al. (2024)	Illumination-Hypothesis Image-Expansion - Attention Region Fusion - Long Short-Term Memory (LSTM) - Deep Learning-Based Object Detector	Predictive Learning Robotic disassembly
Bassiou et al. (2021)	YOLOv5x	Laptops
Yildiz et al. (2020)	DBSCAN, HDBSCAN	Computer hard disks
Yildiz and Wörgötter (2019)	DCNN - Hough transform	
Rehnholm (2021)	CNN - YOLOv4	Battery Pack
Biehler et al. (2023)	3D point cloud transfer learning with contrastive augmentation for robust and domain-invariant defect detection	Electronic component
Zheng et al. (2022)	PointNet-based 3D deep neural network for automatic recognition of mechanical parts in disassembly tasks	Automotive component identification

### 4.4 Safety standards for collaborative applications

The use of robotic disassembly systems is rapidly becoming an established feature across a range of industries, providing efficiency and accuracy in the disassembly of electronic devices and machinery. However, safety of human operators and the environment has become a major issue as the use of robotic systems for disassembly tasks continues to grow. The integration of safety measures plays a key role in minimising the risks involved in these tasks. ISO 10218 is a major standard governing the safety aspects of industrial robots, particularly those used in disassembly applications.

#### 4.4.1 ISO 10218-1:2011 - safety requirements for industrial robots—part 1: robots

This section deals with the essential safety aspects of industrial robots, including robot design, system integration and installation. Guidelines are given for risk assessment, safeguards, and implementation of safety features to avoid potential accidents in normal working conditions as well as under exceptional conditions (ISO, 2024a).

#### 4.4.2 ISO 10218-2:2011 - safety requirements for industrial robots—Part 2: robot systems and integration

In the second part, the standard deals with the interaction between a robot and its environment, which includes human workers. This part of the standard specifies collaborative operation and describes the safety measures that need to be taken when humans are operating in the proximity of robots, focusing on the need to reduce risks (ISO, 2024b).

#### 4.4.3 ISO/TS 15066:2016 - robots and robotic devices—collaborative robots

The technical specification delivers safety guidelines for robots that function together with human workers. Human-robot interfaces must establish the maximum force and pressure levels which humans can tolerate when accidently interacting with robotic systems. Risk assessment procedures and safety measures with guidelines for human-robot interactions are specified in the document while different collaborative operation modes such as speed and separation monitoring and hand guiding and power/force limiting are detailed. The standard functions as an essential addition to ISO 10218 by reducing risks in collaborative environments that combine human operators with robotic systems (ISO, 2024c).

Looking beyond ISO standards, a bunch of organisations have come up with safety guidelines that shape how robots are designed and used. In the EU the Machinery Directive (2006/42/EC) (Eur-Lex, 2006) sets out a legal framework for machine safety including industrial robots which is often linked to EN ISO 10218 and ISO/TS 15066. In other sectors such as defence, safety rules cover autonomous and unmanned systems. NATO’s STANAG 4586 (STANAG 4586, 2025) standard helps to standardise drone operations while the US Department of Defence applies MIL-STD-882E (Department of Defense DoD, 2023) to manage risks associated with complex, high-risk robotic systems. Together, these policies highlight the general need for safety from factories to battlefields in order to ensure both the protection of people and the reliability of systems.

There are four collaborative operative modes identified by robot safety standards as mentioned in Table 9. Safety-rated Monitored Stop is the most basic form of collaboration. In this case, the worker executes manual tasks within the operational space shared by man and robot. Inside this collaboration zone, the human and the robot can work, however not at the same time since the robot is not allowed to move when the operator is occupying this shared space. Such cooperation is ideally suited to the manual placement of objects on the robot end-effector, whether for visual checking, finishing or complex tasks (Vysocky and Novak, 2016). Secondly, Hand Guidance. Also referred to as “direct teach”, it allows the operator in this collaborative mode to teach the robot positions by simply moving the robot, with no need for an additional interface, for example, a robot teach pendant. The robot arm’s weight is balanced to maintain its position. Using a guiding device, which drives the robot’s movement, allows the operator to be in direct contact with the machine (Ogura et al., 2012). Third mode refers to Speed and Separation Monitoring. This mode, also referred to as Speed and Position Monitoring (SPM), provides human access to the robot space using safety monitoring sensors. When a human is in the 1st zone, the robot operates at full speed, in the 2nd zone at slower speed, and in the 3rd zone it stops when the human enters (Marvel, 2013). A fourth mode is Power and Force limitation. It involves limiting the motor’s power and strength, to enable a human worker to work side-by-side with the robot. It requires specific equipment and control models to handle collisions between robot and human without negative consequences for the human (Haddadin et al., 2015). Table 10 demonstrates the application of safety technologies for human-robot collaboration in dynamic environments through key studies. The research presents both techniques and verified outcomes to display progress made in robot collision prevention alongside human perception of safety and robotic-human interface systems. Safety technology approaches highlight how vital it is to combine adaptive and predictive systems for maintaining safety and efficiency in shared work environments.

**TABLE 9:** Four collaborative operating modes specified by robot safety standards.

Level	Technologies
1	Safety-rated Monitored Stop (SMS)
2	Hand Guidance (HG)
3	Speed and Separation Monitoring (SSM)
4	Power and Force Limiting (PFL)

**TABLE 10:** Comparative table of technologies/algorithms used in safety.

Authors	Methods used	Results
Wu et al. (2024)	Position-Heading CBF, Flexible Performance Functions	- Successful collision avoidance in dynamic environments - Ensures safety-critical trajectory tracking with performance guarantees
Wang et al. (2024)	Deep Learning, Semi-Supervised Object Detection	- Real-time monitoring with high detection accuracy - Effective in “cage-free” manufacturing environments
Zhu et al. (2024)	Residual RL Models, Safety Task Design	- Superior collision avoidance in dense crowds - Achieved high success rates in both simulation and real-world experiments
Feng et al. (2024)	Risk-Area Model, Dynamic Collision Avoidance	- Significantly reduced unsafe actions in critical scenarios - Maintains task efficiency despite safety interventions
Sahin and Savur (2022)	Sensitivity Tuning, Velocity and Trajectory Adjustment	- Moderate positive linear relationship between during-trial and after-trial safety ratings - Significant improvement in perceived safety when sensitivity or all behaviors (velocity, trajectory, sensitivity) are adjusted

### 4.5 Supplementary trends

Self-supervised learning refers to an approach based on a machine learning concept in which a model can learn representations directly from the data itself, with no explicit supervision. In conventional supervised learning, the model learns from labeled data, where every input is associated with a corresponding target output. Yet in self-supervised learning, a model is trained to predict certain aspects of the data without depending on external labels.

#### 4.5.1 Grasp2Vec

It combines the analysis of robotic grasping with the integration of words, presenting grasping actions as vectors like words in NLP. This exploits the properties of vector space for tasks such as grasp recommendation and similarity analysis, providing a new approach to improving robotic manipulation capabilities (Jang et al., 2018). As presented by Jang et al. (2018), there’s a representation learning from input, using a robotic arm to remove an object from the scene and examine the resulting scene and the object in the gripper. Making sure that the difference between the representations of the scene corresponds to the representation of the object. Also, as supervision of grasping using learned representations, A similarity metric between object representations has been used as a reward for grasping an object, which eliminates the need to manually label the results of the grasp.

The grasping system detects motion while operating objects yet remains unaware of which specific objects it handles. The system includes cameras that record imagery of both the complete scene and the target object during gripping operations. During the initial training, the grasping robot is run to grasp any object at random, producing a triplicate of images (Spre, Spost, O): The camera shows O as an image representation of what the camera detected. The scene before the capture shows the object at position O. The image Spost shows the captured scene after capture while O is absent from the image.

$$LGrasp2Vec=NPairs\left(\left(\varphi s\left(spre\right)-\varphi s\left(spost\right)\right),\varphi o\left(o\right)\right)+NPairs\left(\varphi o\left(o\right),\left(\varphi s\left(spre\right)-\varphi s\left(spost\right)\right)\right)$$

#### 4.5.2 RIG (reinforcement learning with imagined goals)

RIG combines reinforcement learning with self-supervised learning methods through an integrated system. The addition of imagined goals improves sample efficiency and policy robustness through exploration and learning of generalized policies. The method has demonstrated utility across different domains to improve RL agent performance within complicated scenarios.

Like grasp2vec, RIG also applies data augmentation through latent relabeling of targets: specifically, half of the targets are randomly generated from the *a priori* and the other half are selected using HER. As with grasp2vec, the rewards do not depend on ground truth states, but only on the learned state encoding, so it can be used for training on real robots as outlined in the work of Nair et al. (2018).

#### 4.5.3 TCN (time-contrastive networks)

TCN (Time-Contrastive Networks) is based on the intuition that different viewpoints of the same scene at the same time should share the same integration (as in FaceNet), whereas the integration should vary over time, even for the same camera viewpoint. Therefore, the integration captures the semantic meaning of the underlying state rather than visual similarity. TCN integration is trained with triplet loss. Within the work of Sermanet et al. (2018), training data are collected by simultaneously taking videos of the same scene, but from different angles. All videos are unlabeled.

The blue frames selected from two camera views at the same timestep are anchor and positive samples, while the red frame at a different timestep is the negative sample. TCNs are also used in various sequential data tasks such as speech recognition, natural language processing and time series prediction. They have demonstrated competitive performance against other recurrent and convolutional architectures, particularly for tasks requiring long-term dependencies and the capture of complex temporal patterns.

#### 4.5.4 SOAR cognitive architecture

A powerful framework designed to emulate human cognitive processes and decision-making capabilities in complex and dynamic environments. As outlined in recent studies, Soar integrates state-operator-action-result (SOAR) reasoning to systematically analyze and respond to environmental stimulus. It operates by constructing state spaces that combine long-term memory elements (domain-specific knowledge) with short-term memory elements (real time environmental data). This architecture supports the generation of interpretable decisions through rule-based mechanisms, allowing agents to adaptively transition between states to achieve predefined goals The decision-making process in Soar can be expressed as:

$$\text{Decision}=\arg\max_{O\in O}\;\left(P\left(O\right)+R\left(O\right)\right)$$

where O represents an operator from the set of available operators $O,P\left(O\right)$ denotes the preference value of operator O , and $R\left(O\right)$ is the reinforcement-based reward associated with selecting O . Soar evaluates all operators, selecting the one with the highest combined preference and rewarding them to achieve efficient and goal-oriented transitions between states. Soar’s unique learning mechanisms enhance its adaptability. Through the chunking process Soar extracts new rules from historical problem-solving situations which shorten future decision times. Decision making benefits from reinforcement learning which adjusts operator preferences through feedback gathered from past activities. Through its episodic memory Soar can examine historical situations alongside present circumstances to generate decisions even when no direct rules exist.

#### 4.5.5 Adaptive control of thought-rational (ACT-R)

The robust cognitive architecture shows exceptional ability to model human thinking through its integration of perception modules with motor execution and memory retrieval systems. ACT-R contains two memory modules with procedural and declarative functions which operate together through buffers and pattern-matching to execute behavior-controlling production rules. The robotics systems utilizing ACT-R have shown successful deployment for adaptive tasks that support human-robot interaction along with collaborative functions. The system achieves performance through the combination of real-world sensory data with established cognitive models that produce context-specific responses. The modular design of ACT-R enables advanced perception along with motor functions that benefit humanoid robot implementations such as Pepper. Robotics systems that use ACT-R processing generate human emotion comprehension abilities which enable them to modify verbal and non-verbal outputs for improved human robot interaction. Practical implementations of ACT-R prove its ability to merge robotic operational competencies with human cognitive operations thereby developing more empathetic robotic platforms.

#### 4.5.6 Robot operating system (ROS)

As middleware frameworks the Robot Operating System and its successor ROS2 are the key to how distributed robotics components talk and work together. It makes hardware complexity easier to deal with through a publish-subscribe model using topics, services and messages making it simpler to build robotics features in a modular way. While ROS1 is relying on a centralized master node, ROS2 adopts a decentralized approach based on the Data Distribution Service (DDS) offering better scalability and real-time communication also as fault tolerance. Both systems serve as the foundation for robotics software integration which support not only task orchestration but also system-level extensions such as safety monitoring as demonstrated by recent efforts (Rivera et al., 2020).

## 5 Conclusion and perspectives

In this paper, an in-depth systematic review is summarized on artificial intelligence approaches for robotic disassembly. Focusing on the optimization and strategic planning methodologies of HRC, CV, and safety measures. The benefits in these technologies are enormous, showing their potential to improve overall efficiency, precision, and flexibility in disassembly processes. Integration of machine learning, robotic handling, and advanced sensor systems finally seems to produce promising results toward disassembly tasks automation. Such technologies adequately treat issues related to the e-waste material diversity and intricate product designs, while being dependent on systems that will be totally safe for human operators, user-friendly, and easy to deploy. Despite all these advances, there still exist a few barriers to preventing the widespread robotic disassembly. Key issues are cost-effective implementation, scalability, and legal issues, these all must be addressed for proper application of these technologies in an industrial setup. More research and development need to be done further to give solutions related to these challenges, as this will move the field forward. There would need to be further advancement as well, in synthesis with robotics engineering, AI research, and policymaking. This form of interdisciplinary collaboration will be a defining feature in the future landscape for robotic disassembly. Such synchronization between technologic advancements, on one side, and practical, economic, and regulatory frameworks, on the other, could place the preconditions for effective and widespread introduction of robotic disassembly systems, which in turn will be an eventual step in the direction of more sustainable and efficient practices in e-waste management.

## Data Availability

The original contributions presented in the study are included in the article/supplementary material, further inquiries can be directed to the corresponding author.
